# Mr. Ring, on Vaccine Inoculation

**Published:** 1802-04

**Authors:** John Ring

**Affiliations:** New Street, Hanover Square


### *To the Editors of the Medical and Physical Journal.*

GENTLEMEN,

THE rapid progress which Vaccine Inoculation is still making, cannot but afford the most sincere satisfaction to every benevolent mind. Advices are received from Turkey, that an infant of the favourite of the Grand Segnior has undergone this salutary process. From Russia we hear, that another crowned head also patronizes this improvement of the healing art. The Empress Dowager has ordered the first child who was inoculated with Vaccine virus to be christened Vaccinoff; and settled a pension on her.

I am also informed by the Rev. Mr. Pitt, Chaplain to the English Factory in Russia, that this illustrious personage countenances the practice. It is a little remarkable, that after repeated failures, the new inoculation succeeded at Moscow while the Court was there; and, at the same time, at Petersburg. From these two sources it is now become general.

Mr. Pitt adds, that although this practice is every where of great importance, it is, perhaps, no where so much so as in Russia; which he attributes to the habits of the Russian peasants. A nobleman who has very large estates assured him, that throughout his villages, the ravages of the Small-pox are little inferior to those of the plague.

To these communications on a popular topic I beg leave to add a few others, which, I flatter myself, will not prove uninteresting.

It is well known, that in cases of Ranula, simple incision frequently effects a cure. In some instances, however, and particularly where the cyst is thick, this method is insufficient.

A case of this kind occurred a few months ago; in which a surgeon had five times operated in this manner, but without success. When the child was brought to me, the tumor was so large, that it compressed the tongue with great force against the the roof of the mouth, fo as totally to impede fucking, and even deglutition. In this extremity, the excifion of a portion of the cyft afforded prefent relief, and produced a perfect cure.

The next fubject on which I fhall offer a few remarks, is the treatment of the Itch; a difeafe which is fufficiently afflicting and difgufting, without the aid of that infernal drug, Brimftone.

Having been frequently difappointed in my attempts to cure it with ointment of white precipitate, and folutions of fublimate, and having obferved that the latter caufed confiderable inflammation of the fkin, without proving a certain remedy for the diftemper, I combined the two with an unctuous fubftance; and, from that time, about five and twenty years, have never known it fail in a fingle inftance. The form is as follows:

℞. Hydrarg. muriat. gr. x. Calcis hydrarg. alb. ʒj. Adipis fuill. ℥iij. Effent. bergamot. ʒj. M. ft. unguentum.

With this ointment the patients are to rub themfelves all over, every night. The quantity here prefcribed muft not be ufed in lefs than ten nights, even in an adult. Care muft be taken, not to rub too much on any particular part, efpecially on the bend of the elbow; otherwife, inflammation and excoriation may be the confequence.

The abfurd cuftom of giving fulphur, or any other medicine, internally, for this cutaneous diforder, ought to be difcontinued. I have often known dyfenteries occafioned by fuch practices; and the pretended remedy prove worfe than the difeafe.

The preparation of mercury here propofed feldom affects the falivary glands. Its greateft inconvenience is a troublefome heat, rafh, or other effects of too powerful a ftimulus applied to the fkin; which, if the patient be properly cautioned, may be inftantly relieved, by fubftituting a little plain lard, inftead of the ointment. This application ought to be continued, till the troublefome fymptoms are perfectly removed.

A preparation of the fame kind, but only of half the ftrength of that already prefcribed, is one of the moft efficacious remedies for the Tinea Capitis; in the cure of which, conftant care muft be taken to keep the furrounding hair as fhort as poffible. By means of this ointment, applied once a day, I have fucceeded in the cure of every cafe in which it has been tried, amounting to many hundreds. One of the patients had laboured under the difeafe fourteen years; and his father wifely dertermined to get him cured, if poffible, before he put him out as an apprentice.

Many others have been cured by the fame remedy, who had been given up as incurable. No internal medicine whatever was was exhibited in any of thefe cafes; a fufficient proof that no internal medicine is neceffary in fuch cafes, whatever may be the prejudice, or whatever may be the intereft, of medical men.

The fame topical application proves equally efficacious in the cafe of tetters, and various other affections of the fkin.

Should thefe obfervations be deemed worthy of infertion, I fhall ere long add to their number.

*New Street, Hanover Square,* I am, &c.

*March* 16, 1802. JOHN RING.

